# Orientation Tuning Depends on Spatial Frequency in Mouse Visual Cortex

**DOI:** 10.1523/ENEURO.0217-16.2016

**Published:** 2016-09-28

**Authors:** Inbal Ayzenshtat, Jesse Jackson, Rafael Yuste

**Affiliations:** NeuroTechnology Center, Department of Biological Sciences, Columbia University, New York, New York 10027

**Keywords:** visual cortex, cortical tuning, orientation selectivity, spatial frequency, two-photon, calcium imaging, mouse, asymmetry, Gabor model

## Abstract

The response properties of neurons to sensory stimuli have been used to identify their receptive fields and to functionally map sensory systems. In primary visual cortex, most neurons are selective to a particular orientation and spatial frequency of the visual stimulus. Using two-photon calcium imaging of neuronal populations from the primary visual cortex of mice, we have characterized the response properties of neurons to various orientations and spatial frequencies. Surprisingly, we found that the orientation selectivity of neurons actually depends on the spatial frequency of the stimulus. This dependence can be easily explained if one assumed spatially asymmetric Gabor-type receptive fields. We propose that receptive fields of neurons in layer 2/3 of visual cortex are indeed spatially asymmetric, and that this asymmetry could be used effectively by the visual system to encode natural scenes.

## Significance Statement

In this manuscript, we show that the orientation selectivity of neurons in primary visual cortex of the mouse is highly dependent on the stimulus spatial frequency (SF). This dependence is demonstrated quantitatively by a decrease in the selectivity strength of cells in nonoptimum SF, and, more importantly, it is also demonstrated qualitatively by a shift in the preferred orientation of cells in nonoptimum SF. We show that a receptive-field model of a 2D asymmetric Gabor, rather than a symmetric one, can explain this surprising observation. Therefore, we propose that the receptive fields of neurons in layer 2/3 of mouse visual cortex are spatially asymmetric, and this asymmetry could be used effectively by the visual system to encode natural scenes.

## Introduction

Neurons in the primary visual cortex (V1) have been traditionally described by their receptive field (RF) structure and their response characteristics (Hubel and Wiesel, 1962; Skottun et al., 1991). They are classified into two major groups, simple and complex, and exhibit spatially localized receptive fields that consist of distinct elongated ON and OFF subfields. In mouse visual cortex, ∼75% of the orientation-selective neurons in layer 2/3 are classified as simple cells, showing response characteristics similar to simple cells in visual cortex of monkeys or cats (Niell and Stryker, 2008).

To capture the selectivity of a neuron to a certain feature of a visual stimulus (e.g., orientation, spatial frequency (SF), size, position, and speed), it is convenient to measure one-dimensional (1D) tuning curves that show the average response of a neuron to a specific feature value. Customary tuning models propose that the response strength of a neuron can be predicted based on the similarity between the optimal stimulus of a neuron and the given stimulus. From the 1D tuning curves, which describe the response behavior to one stimulus feature, one can calculate several parameters such as the preferred stimulus, the strength of the selectivity, or the width of the selectivity that quantifies the specificity level of the neuron. In visual cortex, for example, since neurons are highly responsive to lines and edges, such curves, which commonly characterize simple cells in V1, are the orientation–tuning and spatial frequency–tuning curves. However, reducing the complexity of the RF spatial structure to 1D tuning curves in order to study individual features, comes at a cost of losing information that might be critical for understanding the neuronal representation of sensory information. Here, we measured the population responses of neurons in L2/3 of mice V1 to drifting gratings that varied in both orientation and SF. We used *in vivo* two-photon Ca^2+^ imaging to measure evoked responses from hundreds of V1 neurons. Accordingly, we calculated a two-dimensional (2D) tuning matrix, and studied the relationship between orientation and SF selectivity. Then, we compared orientation–tuning curves in various SFs. First, we found that the orientation selectivity of a neuron depends strongly on the stimulus SF, such that when we presented gratings with higher or lower SFs than the optimum, the orientation selectivity was significantly reduced. In addition to a quantitative change in the selectivity strength of the neurons, we also observed a qualitative change in the preferred stimulus of the neuron. As we moved away from the optimal SF, to either lower or higher SFs, there was a significant shift in the preferred orientation (Pref) of the neurons. Dependence between orientation and SF selectivity of cells has been previously suggested in the visual cortex of primates and cats (Andrews and Pollen, 1979; Vidyasagar and Sigüenza, 1985; Webster and De Valois, 1985; Jones et al., 1987; Zhu et al., 2010).

In order to explain this dependence between orientation selectivity and SF, we used the common Gabor model (Gabor, 1946) to predict the neuronal response to various stimuli. A Gabor filter is a Gaussian modulated sinusoid, which well describes the receptive fields of simple cells and successfully models their responses (Marcelja, 1980; Field and Tolhurst, 1986; Jones and Palmer, 1987b). However, the classic Gabor model, even though it succeeds in predicting multiple neuronal responses, cannot capture the full variety and complexity of the visual system. And, indeed, we found that the classic mathematical description of a 2D symmetric Gabor model (with either odd or even amplitude symmetry) cannot account for our experimental findings. However, spatially modifying the classic model to introduce a 2D asymmetry by way of tilting the Gabor against its elongated axis generates a fundamental change in the response predictions, which qualitatively explains our experimental observations.

The modified Gabor model presented in this article can explain the response characteristics of a population of neurons and suggests that the receptive field of many cells in layer 2/3 of visual cortex of mice demonstrates a central asymmetry in its 2D spatial organization.

## Materials and Methods

### Animals

Animal handling and experimentation were performed in accordance with the National Institutes of Health and Columbia University institutional animal care guidelines. Animals of both sexes were used and were housed in a temperature-controlled environment on a 12 h light/dark cycle. We used a total of five mice, either WT or VIP-Cre crossed with LSL-tdTomato [postnatal day 40 (P40) to P80; The Jackson Laboratory].

### Surgery

The mice were placed on a warming plate (37ºC) and anesthetized with isoflurane (initially 2%, and reduced to 1–1.5% during surgery) administered via nose cone. A custom-made titanium head plate was attached to the skull using dental cement. Subsequently, a craniotomy (∼1 × 1 mm) was made over the primary visual cortex (3.5–4.5 mm posterior to bregma, 2.3–2.7 mm lateral to midline; putative monocular region) using a dental drill (Osada). An ophthalmic ointment was applied on both eyes to protect the eyes from dehydration during surgery, and was removed during visual stimulation.

### Dye loading

For bulk loading of cortical neurons, Oregon Green Bapta-1 (OGB-1) AM (Molecular Probes) was first mixed with 4 μl of pluronic acid (20% in DMSO) and further diluted in 35 μl of dye buffer (150 mm NaCl, 2.5 mm KCl, and 10 mm HEPES, pH 7.4). Sulforhodamine-101 (SR101; Molecular Probes) at a concentration of 50 μm was added to the solution to label astrocytes (Nimmerjahn et al., 2004). Animals were head fixed, and the dye was slowly pressure injected into the left visual cortex at a depth of ∼130–200 μm below the dura surface (layer 2/3) at an angle of 30º through a patch pipette (outer diameter, ∼1–2 μm) using a Picospritzer II. Two to four injections were carried out at 10 psi for 8 min, each under visual control of a two-photon imaging microscope [850 nm excitation; 10× water-immersion objective; 0.5 numerical aperture (NA); Olympus]. After dye injections, the exposed cortex was covered with agarose (1.5–2%; Sigma-Aldrich) and a cover glass (World Precision Instruments) to reduce brain motion. Data collection began 60–90 min after injections to ensure dye uptake across a large number of cells. During data collection, light anesthesia was maintained by isoflurane (0.8–0.9%) administered via nose cone (KOPF Instruments). Heart rate, respiration, and oxygen saturation were monitored throughout the experiments using MouseOx (STARR Life Sciences Corp), and the respiration rate was used to monitor and control anesthesia levels.

### Two-photon Ca^2+^ imaging

Imaging was performed with a two-photon moveable objective microscope (Sutter Instrument) and a mode-locked dispersion-precompensated Ti:sapphire laser (Chameleon Vision II, Coherent). Frames were scanned through a 20× (0.95 NA; Olympus) or 25× (1.05 NA, Olympus) water immersion objective. Laser intensity was controlled via Pockels cell (Conoptics) and ranged between 20 and 70 mW. Scanning and image acquisition were controlled using Mscan (4.07 frames/s for 512 × 512 pixels; Sutter Instrument). OGB-1 fluorescence was excited at 950 nm. Fluorescence changes collected with a 20× objective typically varied between 5% and 50%, and between 10% and 70% with a 25× objective. Emission was collected using green (535/50 nm) and red (610/75 nm) filters (Chroma) simultaneously on two photomultiplier tubes.

### Visual stimulation

Visual stimuli were generated in MATLAB (RRID:SCR_001622) using Psychophysics toolbox (Brainard, 1997; Pelli, 1997; Kleiner et al., 2007) and displayed on a gamma-corrected LCD monitor (Dell; 19 inches, 60 Hz refresh rate) positioned 15 cm from the contralateral eye, at ∼45º to the long axis of the animal (spanning ∼114º vertical by ∼140º horizontal of visual space). The presentation of visual stimuli was synchronized with image acquisition using Mscan (Sutter Instrument) and a routine written in MATLAB, such that each stimulus presentation was triggered on the beginning of frame acquisition. The actual time of stimulus presentation was detected with a silicon photodiode (Hamamatsu) attached to the bottom right corner of the screen.

**Figure 1.**
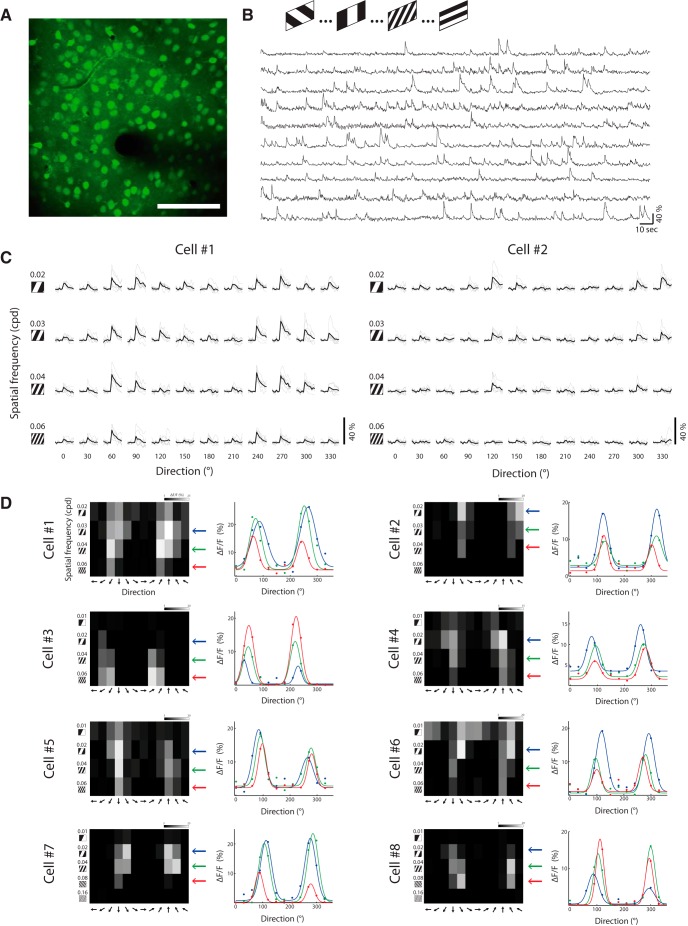
*In vivo* imaging of visual-evoked responses of layer 2/3 neurons. ***A***, Top, A two-photon image (maximum projection) from L2/3 neurons in mouse primary visual cortex, loaded with OGB-1. Scale bar, 100 μm. ***B***, Example traces of Ca^2+^ signals from 10 cells during the presentation of drifting gratings with 4 different spatial frequencies and 12 directions. ***C***, Ca^2+^ responses of two neurons, displayed as a matrix of all stimulus conditions. Columns indicate the direction of motion of the gratings, and rows indicate their SF. Each trial is shown in gray (*n* = 7); average response across trials of a given stimulus is shown in black. ***D***, Tuning matrix of eight cells (cells 1 and 2 are shown in ***C***). Left, Response matrices evoked by each stimulus. Pixels intensity corresponds to the average Δ*F*/*F* over two frames poststimulus presentation and over seven repetitions. Right, Direction–tuning curves fitted with a double Gaussian, measured in various SFs. Colors correspond to SFs marked with arrows next to the matrices on the left.

We presented square-wave drifting gratings (100% contrast) for 670 ms, followed by 3–5 s of uniform gray background (the mean luminance of the gratings) plus a blank condition. The gratings orientation was perpendicular to the drift direction. Gratings were presented at 12 directions of motion (in 30º steps), three sets of four or five spatial frequencies: [0.02, 0.03, 0.04, 0.06 cycles per degree (cpd)], [0.01 0.02 0.04 0.06 cpd], or [0.01 0.02 0.04 0.08 0.16 cpd], and a temporal frequency of 1.5 Hz. All stimuli were block randomized and repeated 5–10 times, and the initial phase of the drifting gratings was kept constant across all trials. Stimuli were presented in a pseudorandom order, but time courses are shown after sorting (Fig. 1C).

### Data analysis

#### Image analysis

All data analyses were performed using built-in and custom software written in MATLAB (MathWorks). Images were first converted to TIFF format and registered to correct for *x*–*y* motion using Turboreg plug-in in ImageJ (Thévenaz et al., 1998; RRID:SCR_014308). Regions of interest (ROIs) were drawn around each cell using a semiautomated algorithm based on fluorescence intensity (mean projection), florescence change (SD projections), and cell size and shape, and were adjusted by visual inspection. Glia cells were excluded from further analysis using SR101 staining, which selectively labels astrocytes in rodent neocortex (Nimmerjahn et al., 2004). Pixels were averaged within each ROI for each image frame. Baseline Ca^2+^ fluorescence was computed for each trial as the mean over 2 s prestimulus. Then, fluorescence values were converted to the percentage change above baseline [response amplitude (Δ*F*/*F*)] according to the following: Δ*F*/*F* = (*F*_1_ − *F*)/*F*, where *F* is the baseline fluorescence and *F*_1_ is the instantaneous fluorescence signal averaged over two frames (∼500 ms) following stimulus onset (*F*_0+2_, *F*_0+3_, where *F*_0_ is the stimulus onset frame).

Responsiveness and reliability criteria were defined as previously described (Marshel et al., 2011). Briefly, neurons were considered responsive if their mean Δ*F*/*F* to any stimulus exceeds 6%. Reliability (δ) was determined according to the following:

$$\delta =\frac{\mu_{max}-\mu_{blank}}{\sigma_{max}-\sigma_{blank}},$$

where *μ*_max_ and *σ*_max_ are the mean and SD of the response to the preferred stimulus, respectively, and *μ*_blank_ and *σ*_blank_ are the mean and SD of the response to the blank stimulus, respectively. Neurons were considered reliable if δ > 1. Only cells that demonstrated visual responsiveness and reliability were chosen for further analysis, which excluded between 38% and 43% of the total number of cells we observed per field of view (FOV). Therefore, we analyzed 85.4 ± 3.6 reliably responsive cells of 156.2 ± 7.67 cells in total (mean ± SEM; *n* = 5 mice).

#### Visual tuning

To calculate tuning curves, we averaged the evoked responses (Δ*F*/*F*) over two frames following stimulus presentation. Then we averaged the response over the number of repetitions (5–10) per stimulus direction (12 directions). Direction tuning curves generated from OGB-1 fluorescence are comparable to those recorded with electrophysiological techniques (Kerlin et al., 2010; Marshel et al., 2011).

Orientation selectivity index (OSI) was computed as follows:

$$OSI=\frac{\mu_{max}-\mu_{orth}}{\mu_{max}+\mu_{orth}},$$

where *μ*_max_ is the mean response to the Pref and *μ*_orth_ is the mean response to the orthogonal orientation (Orth; average of both directions). Only cells that demonstrated an OSI ≥0.3 were chosen for tuning comparisons.

Orientation–tuning curves were fitted with the sum of two Gaussians of identical width, as follows:

$$R\left(\theta \right)=\alpha_{0}+\alpha_{1}e^{\frac{-(\theta -\theta_{0}{)}^{2}}{2\sigma^{2}}}+\alpha_{2}e^{\frac{-(\theta -\theta_{1}{)}^{2}}{2\sigma^{2}}},$$

where *R*(*θ*) is the averaged response to gratings with direction *θ*, and *α*_0_ is the mean Δ*F*/*F* of the four lowest points in the curve. *α*_1_ and *α*_2_ are the amplitudes of the two Gaussians, *θ*_0_ is the preferred direction, *θ*_1_ is the null direction, and *σ* is the SD of the Gaussian function. The sum of two Gaussians fitting was constrained, according to Mazurek et al. (2014), by using several initial conditions, considering the fit with the lowest least square error as the best fit of the data. Peak responses were the maximum Δ*F*/*F* values of the 2D Gaussian fit curve. Half-width at half-height (HWHH) was computed as follows:

$$HWHH=\;\sqrt{2ln2}\sigma ,$$

where *σ* is the SD of the Gaussian function.

To further assess the statistical robustness of the tuning curve fitting, we applied a bootstrap method where we randomly resampled the data with replacement for each cell and obtained a distribution of response values. This procedure was repeated 100 times, where each repetition was fitted with a double Gaussian (Mazurek et al., 2014). This yielded a distribution of values for each of the tuning curve parameters. The mean values of the resampled data were then used for comparing population parameters showing the same statistical significance as achieved by comparing parameters of tuning curves obtained by fitting the data that included all the trials.

Additionally, orientation selectivity was assessed using a metric based on 1 − circular variance (CirVar; Ringach et al., 2002), as follows:

$$1-Cir\,Var=\frac{\sum R\left(\theta \right)\text{exp}(2i\theta )}{\sum R(\theta )}$$

where *R*(*θ*) is the averaged response to gratings with orientation *θ*. This metric takes into account both tuning width and depth of modulation, and was found to be more reliable than extracting parameters following curve fitting, and more sensitive for detecting differences in selectivity between two populations (Piscopo et al., 2013; Mazurek et al., 2014).

#### 2D Gabor model

The two-dimensional Gabor function is the product of a 2D sinusoid wave with a circular Gaussian envelope (see Fig. 3A), which was defined as follows:

$$g\left(x,y\right)=\text{exp}(-\frac{x^{2}+\gamma^{2}{y\prime }^{2}}{2\sigma^{2}})\text{cos}(2\pi \frac{x\prime }{\lambda }+\;\psi ),$$

x′= xcosθ+ysinθ,

y′=−xsinθ+ycosθ,

where *σ* is the SD of the Gaussian envelope, which determines the size of the receptive field; *γ* is the spatial aspect ratio of the Gaussian, which determines the ellipticity of the Gabor (in a circular Gaussian *γ* = 1*)*; *λ* is the wavelength of the sinusoid; *θ* is the orientation; and *ψ* is the phase offset.

In the tilted Gabor model, we introduced another parameter, which is the angle of the Gaussian tilt (*ϕ*) with respect to the sinusoid wave. To generate a tilted Gabor filter, we first generated a symmetric 2D Gaussian with a spatial aspect ratio of γ = 0.5 (see also Fig. 4B) and then applied a tilt by multiplying it with a 2D rotation matrix *A* with *ϕ* = 30º, as follows:

$$A=[\begin{array}{cc} cos\phi & -sin\phi \\ sin\phi & cos\phi \end{array}].$$

#### Calculating the predicted response

To calculate the predicted response, *R*(*I*), of a neuron, we computed the inner product between the RF *f*(*x*,*y*)—either a symmetric or a tilted Gabor filter, and the stimulus *I*(*x*,*y*)—square-wave gratings drifting in 180 different orientations with 1º interval (see Fig. 3B, examples). Since we measured neuronal responses by recording Ca^2+^ signals, we obtained tuning matrices by maximizing the averaged evoked response per stimulus. Therefore, to compute the predicted response, we calculated the inner product per stimulus orientation for each phase of the stimulus between 0 and 2*π* (21 samples, *π*/10 apart; see Fig. 5) and then maximized the response across phase.

## Results

### Layer 2/3 cells in mouse V1 show dependence of orientation selectivity on spatial frequency

In lightly anesthetized mice, we identified cells in layer 2/3 of visual cortex using two-photon Ca^2+^ imaging and monitored the activity of neuronal populations (Marshel et al., 2011; Miller et al., 2014) evoked by a brief visual stimulus presented to the contralateral visual field. We characterized the response dynamics by optically recording Ca^2+^ signals of OGB-1 from cells in upper layer 2/3 (depth, ∼130–200 μm; *n* = 5 animals) in a typical FOV (Fig. 1A,B). Specifically, we measured spatial frequency–tuning and direction–tuning curves of each neuron in our FOV by averaging across trials the response to each stimulus (Fig. 1C,D). Stimuli were square-wave drifting gratings presented at 1.5 Hz for 670 ms (approximately one cycle) and varied across 12 directions and four or five SFs (see Materials and Methods).

**Figure 2.**
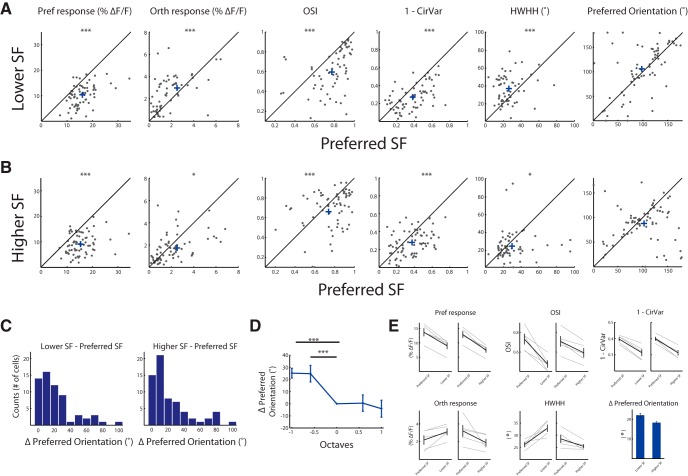
Dependence of orientation tuning curves on SF. ***A***, Comparison of tuning parameters between the preferred SF of each cell and one lower SF. Shown are scatter plots of cells from one network that had preferred SF of ≥0.02 cpd (*n* = 67 cells). Each circle represents a cell, and the cross represents the population average. Shown from left to right are the Δ*F*/*F* to the Pref, Δ*F*/*F* to the Orth, OSI, 1-CirVar, HWHH, and the preferred orientation. (Note that the preferred orientation is determined according to the average response across SFs; therefore, a few cells exhibit a marginally higher Pref response to nonoptimal SFs.) ***B***, Comparison of tuning parameters between the preferred SF of each cell and one higher SF. Scatter plots of cells with preferred SF of ≤0.04 cpd (*n* = 73 cells). Shown are the same parameters as seen in ***A***. ***C***, Histogram of the shift in the preferred orientation with a change in SF. Left, The absolute value of the difference between the lower SF and the preferred SF. Right, The absolute value of the difference between the higher SF and the preferred SF. ***D***, The average shift in the preferred orientation across all the tuned cells (*n* = 83) aligned according to the preferred SF. ***E***, Population mean showing the change in tuning parameters, pooled from five animals. Gray lines depict individual animals, and black lines depict the mean ± SEM across animals.

Initial characterization of the stimulus-evoked responses of the local network showed dependence of the orientation selectivity of the cells on SF. We characterized this dependence by calculating orientation–tuning curves for each SF and then estimating six parameters derived from these curves: (1) response amplitude of the Pref; (2) response amplitude of the Orth; (3) OSI; (4) 1 − CirVar; (5) HWHH; and (6) the preferred orientation (see Materials and Methods). For each cell (85.4 ± 3.6 cells/animal, mean ± SEM), we compared its tuning curve at the preferred SF, with the tuning curves at SFs below (Fig. 2A) and above (Fig. 2B) the preferred SF.

Tuning curves at SFs below the preferred SF had by definition reduced responses to the preferred orientation (*p* < 10^−10^, *n* = 67 cells; Wilcoxon signed-rank test), but also showed increased responses to the orthogonal-to-preferred orientation (*p* < 0.001, Wilcoxon signed-rank test), a decrease in OSI (*p* < 10^−7^, Wilcoxon signed-rank test), a decrease in 1 − CirVar (*p* < 10^−6^, Wilcoxon signed-rank test), and an increase in HWHH (*p* < 10^−4^, Wilcoxon signed-rank test; Fig. 2A). In addition, we observed that some cells had a significant shift in their preferred orientation (Fig 2A, right; since we sampled 12 directions between 0º and 360º, for this analysis we subtracted 180º from the preferred direction of cells that had preferred direction >180º). To quantify this shift, regardless of its sign, we calculated the absolute value of the Δ in preferred orientation between the preferred SF and SF below the preferred. We observed a mean shift of a 22.9° ± 2.8º (mean ± SEM; Fig. 2C, left).

Tuning curves measured with SFs above the preferred SF had a decreased response to the preferred orientation (*p* < 10^−11^, *n* = 73 cells; Wilcoxon signed-rank test); and showed a slight but significant decrease in the orthogonal-to-preferred responses (*p* < 0.01), a decrease in OSI (*p* < 0.01, Wilcoxon signed-rank test), a decrease in 1 − CirVar (*p* < 10^−4^, Wilcoxon signed-rank test), and a slight decrease in HWHH (*p* < 0.05, Wilcoxon signed-rank test). Here too, we found cells with a significant shift in their preferred orientation (Fig. 2A, right), and, indeed, the Δ in preferred orientation was 18.5 ± 2.5º (mean ± SEM; Fig. 2C, right). Further analysis of the change in the preferred orientation in nonoptimal SFs revealed a monotonic averaged shift from SF below the preferred orientation to SF above the preferred orientation, which was quantified as the averaged shift across cells shown in Figure 2, C and D, and exemplified in the tuning curves of single cells shown in Figure 1D. The population results shown in Figure 2, A and B, were consistent across animals. The preferred response was decreased, as expected, at lower and higher SFs (Δ4.73 ± 0.74% and Δ5.39 ± 0.66%, respectively; *n* = 5 animals; 85 ± 3.6 cells; *t* test, *p* < 0.005); the Orth response was increased and decreased at lower and higher SFs, respectively (Δ0.94 ± 0.31% and Δ1.32 ± 0.67%, respectively; *t* test, *p* < 0.05); the mean OSI was significantly decreased at lower and higher SFs (Δ0.26 ± 0.05% and Δ0.12 ± 0.02%, respectively; *t* test, *p* < 0.01); the mean (1 − CirVar) was significantly decreased at lower and higher SFs (Δ0.084 ± 0.010% and Δ0.087 ± 0.009%, respectively; *t* test, *p* < 0.01); the HWHH was increased and decreased at lower and higher SFs, respectively (Δ6.7 ± 1.7% and Δ3.1 ± 1.1%, respectively; *t* test, *p* < 0.05), and the preferred orientation had a mean shift of 22.1 ± 0.9º at a lower SF and a 18.3 ± 0.6º shift at a higher SF (mean ± SEM; Fig. 2E).

### A tilted Gabor model explains a shift in the preferred orientation at different SFs

**Figure 3.**
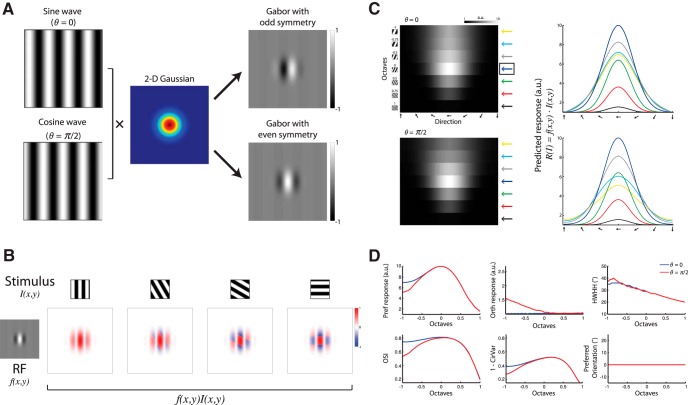
Predicting the responses of a simple cell based on an RF model of a 2D symmetric Gabor model. ***A***, A 2D-oriented Gabor function: a sinusoidal plane wave weighted by a Gaussian envelope in two different phases: *θ* = 0 (sine wave) or *θ* = π/2 (cosine wave) shown at the top and bottom panels that generate Gabor filters with odd and even symmetry, respectively. ***B***, Constructing spatiotemporally oriented impulse responses from gratings stimuli drifting against a Gabor RF. Top, Examples of stimuli with four different orientations. Bottom, Left, An example of a Gabor RF with *θ* = π/2. Bottom, Right, Four examples where each box depicts the overlap of the grating stimulus crossing the RF in a phase that yields the maximum response, calculated as the inner product between the stimulus and the RF. ***C***, A predicted response tuning matrix computed as the inner product between the Gabor RF model shown in ***A***, and stimuli of square-wave gratings with various orientations (1º interval) and seven SFs. Each pixel represents the inner product between the RF and the stimulus (maximized across phase, see Materials and Methods). The row marked with a blue arrow on the right denotes the preferred SF and was taken as a reference for comparing other SFs. Right, Orientation tuning curves at various SFs, color coded according to the arrows shown next to the predicted tuning matrix. Top and bottom panels correspond to RF with *θ* = 0 and *θ* = π/2, respectively. a.u., Arbitrary units. ***D***, Comparison of orientation tuning parameters between various SFs, based on the predicted response shown in ***C***. Blue and red lines depict the parameters calculated based on a Gabor RF model with *θ* = 0 and *θ* = π/2, respectively. Shown are the Δ*F*/*F* to the Pref, the Δ*F*/*F* to the Orth, OSI, 1 − CirVar, HWHH, and the preferred orientation.

**Figure 4.**
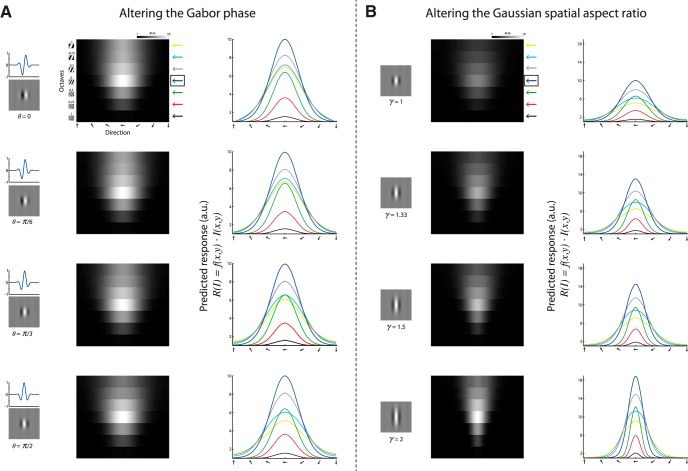
Altering the Gabor model parameters for predicting neural responses. ***A***, A predicted response tuning matrix computed as the inner product between the Gabor RF model shown in Figure 3 and stimuli of square-wave gratings with various orientations (1º interval) and seven SFs. Each pixel represents the inner product between the RF and the stimulus (maximized across phase). The row marked with a blue arrow on the right denotes the preferred SF and was taken as a reference for comparing other SFs. Right, Orientation–tuning curves at various SFs, color coded according to the arrows shown next to the predicted tuning matrix. Each panel corresponds to RF with *θ* = 0, *θ* = *π*/6, *θ* = *π*/3, and *θ* = *π*/2. a.u., Arbitrary units. ***B***, Same as in ***A*** only for an RF with *θ* = π/2 and various spatial aspect ratios (*γ*). Each panel corresponds to RF with *γ* = 1, *γ* = 1.33, *γ* = 1.5, and *γ* = 2. Note that these parameter alterations alone could not qualitatively explain a shift in the preferred orientation of the cells at different SFs.

**Figure 5.**
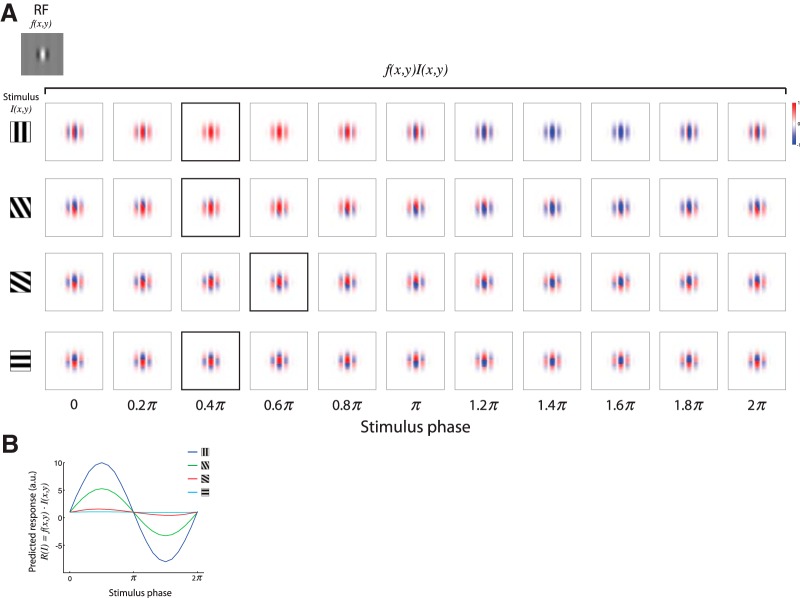
Impulse responses from gratings stimuli drifting against a Gabor RF. ***A***, The impulse responses from gratings stimuli drifting against a Gabor RF in various offsets. Top left, A Gabor RF with *θ* = *π*/2. In each row, on the left is the stimulus presented with one orientation; on the right are 11 examples where each box depicts the overlap of the grating stimulus crossing the RF in a particular phase. The box marked with a black border is the phase yielding the maximum response. ***B***, Bottom, The predicted response as a function of phase, calculated as the inner product between the RF and the stimulus at each phase. The predicted response per stimulus was then maximized across phase.

To our knowledge, the observed shift in the preferred orientation as a function of SF has not been previously reported in mice, although it has been demonstrated in cats (Jones et al., 1987). Therefore, we further investigated whether a known RF model of simple cells in V1 may explain this finding. To do so, we used a Gabor filter model with odd or even symmetry (*θ* = 0, or *θ* = *π*/2 respectively, where *θ* denotes the sine-wave phase; see Materials and Methods; Fig. 3A) to compute the predicted response of a neuron. A Gabor filter with odd symmetry consists of two side-by-side antagonistic regions of equal strength, whereas a Gabor filter with even symmetry demonstrates a central region flanked by two antagonistic regions of equal strength (Fig. 4A). The neuronal response is predicted based on the dot product between the Gabor filter and the stimulus (square-wave drifting gratings). Since we imaged Ca^2+^ signals that have slow dynamics, we could not deduce the optimal phase that each neuron preferred. Therefore, as the experimental tuning curves were calculated based on the maximum evoked responses, we used in our predictions the maximal response across phase (Fig. 5).

We calculated the predicted response for stimuli with various orientations and SFs, and, accordingly, computed the orientation–tuning curves at different SFs (Fig. 3C) and examined the behavior of their parameters (Fig. 3D). First, we observed a significant difference between a Gabor filter with odd symmetry and a Gabor filter with even symmetry in the predicted responses to the orthogonal-to-preferred orientation. This difference also led to a difference in OSI by its definition and in 1 − CirVar. Second, and more importantly, we noticed that a symmetric Gabor filter, regardless of its phase, could not explain a shift in the preferred orientation of a neuron with a change in SF (Fig. 3D). Therefore, we also simulated the predicted response by altering different symmetry components of the Gabor filter [e.g., the phase of the sine wave (*θ*) and the spatial aspect ratio of the Gaussian envelope; *γ*; see Materials and Methods; also see Fig. 4A,B], but these alterations alone could not qualitatively explain a shift in the preferred orientation of the cells.

**Figure 6.**
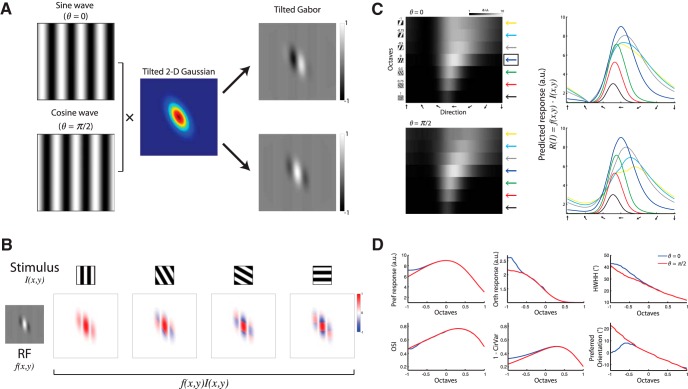
Predicting the responses of a simple cell based on an RF model of a 2D tilted Gabor. ***A***, A 2D tilted Gabor function: a sinusoidal plane wave weighted by a tilted Gaussian envelope (the Gaussian was rotated against the orientation of the sinusoidal plane wave) in two different phases: *θ* = 0 (sine wave) or *θ* = π/2 (cosine wave) shown at the top and bottom panels, respectively. ***B***, Constructing spatiotemporally oriented impulse responses from gratings stimuli drifting against a tilted Gabor RF. Top, Examples of stimuli with four various orientations. Bottom, Left shows an example of a tilted Gabor RF with *θ* = π/2. On the right, four examples where each box depicts the overlap of the grating stimulus crossing the RF in a phase that yields the maximum response, calculated as the inner product between the stimulus and the RF. ***C***, A predicted response tuning matrix computed as the inner product between the tilted Gabor RF model shown in ***A***, and stimuli of square-wave gratings with various orientations (1º interval) and seven SFs. Each pixel represents the inner product between the RF and the stimulus (maximized across phase). The row marked with a blue arrow on the right denotes the preferred SF and was taken as a reference for comparing other SFs. Right, Orientation tuning curves at various SFs, color coded according to the arrows shown next to the predicted tuning matrix. Top and bottom panels correspond to RF with *θ* = 0 and *θ* = π/2, respectively. ***D***, Comparison of orientation–tuning parameters between various SFs, based on the predicted response shown in ***C***. Blue and red lines depict the parameters calculated based on a tilted Gabor RF model with *θ* = 0 and *θ* = π/2, respectively. Shown are the Δ*F*/*F* to the Pref, the Δ*F*/*F* to the Orth, OSI, 1 − CirVar, HWHH, and the preferred orientation.

Since the classic Gabor model did not predict our experimental findings, we sought to examine alternative models. We found that one simple modification to the traditional model could explain the unexpected dependence of orientation on SF. We introduced an asymmetry by way of tilting the Gaussian envelope along the elongated axis of the 2D sine wave (Fig. 6A) and generated a filter that demonstrates a displacement of one subfield relative to the other along the RF orientation axis. We refer to this model as a tilted Gabor. Again, we generated two types of tilted Gabors according to the phase of the sine wave (*θ* = 0, or *θ* = *π*/2) and then predicted the neuronal responses accordingly (Fig. 6B). We examined the dependence of orientation–tuning curves on SF (Fig. 6C) and found that using this modified RF we can predict the behavior of all parameters: a change in the Orth response, a change in OSI, a change in 1 − CirVar, a change in HWHH, and most importantly, a shift in the preferred orientation of the neuron. Here too we observed a difference in the preferred orientation shift between RF phases, such that RF with *θ* = 0 showed a monotonic decrease with the increase in SF, whereas a tilted Gabor with *θ* = *π*/2 showed a nonmonotonic behavior (Fig. 6D).

Collectively, the modified tilted Gabor model better explains our experimental data and suggests an asymmetric 2D organization of the RF ON and OFF subfields.

## Discussion

In this study, we measured neuronal responses from layer 2/3 of primary visual cortex of mice presented with drifting gratings of various orientations and SFs. We observed a unique dependence of orientation–tuning curves on SF, which suggests that the receptive field of some cells in mouse V1 present spatial asymmetry in their RF structural organization.

### Investigating tuning curves in populations of neurons using calcium imaging

Both optical and electrical recording techniques enable the monitoring of activity from hundreds of neurons simultaneously. Therefore, to conduct feasible experiments, one cannot tailor the repertoire of stimuli to the optimal stimulus of each and every neuron. Therefore, the number and the complexity of the experimental stimuli are reduced. In the visual cortex, one common way to reduce the stimulus dimensionality is to measure 1D tuning curves, which are composed of neuronal responses evoked by presenting drifting gratings of various orientations (typically, 8 or 12). This is based on the assumption that the 2D spatial structure of the receptive field can account for a large fraction of the orientation selectivity of simple cells (Lampl et al., 2001). However, orientation–tuning curves measured with a small number of points are just a 1D reduction of the receptive field of a neuron, which has a more complex structure in a high-dimensional space. As a result of this dimensionality reduction, we not only “pay” a quantitative price of reduced neural responses due to nonoptimal stimulus, but we also pay a qualitative price, manifested as a shift in the preferred orientation of the cells in nonoptimal SFs that likely arises from an asymmetric RF structure.

### Cortical neurons have an asymmetric RF spatial structure

The RF structure of a neuron in visual cortex describes the organization of ON and OFF subfields in visual space, and accordingly explains which visual features a neuron is sensitive to. Simple cells in primate and cat cortex have been found to present mainly 1D symmetric organization (i.e., odd-phase or even-phase symmetry along the *x*-axis, orthogonal to the preferred orientation), although there are studies showing that cells do not demonstrate only odd or even symmetry but also exhibit, to a smaller extent, various phases, which breaks the 1D symmetry that exists between the relative strength of each subfield (Kulikowski and Bishop, 1981; Jones and Palmer, 1987a; Ringach, 2002).

However, breaking the amplitude symmetry alone cannot explain the behavior of the observed tuning parameters (Fig. 4). Only a 2D spatial asymmetry that introduces a shift in the relative location of the subfields can predict our experimental findings. In fact, the critical characteristic in predicting a shift in the preferred orientation is the displacement of one RF-subfield relative to the other along the orientation axis, such that it breaks the symmetry in the orthogonal axis. This 2D displacement can be easily modeled by introducing a tilt in the 2D Gaussian envelope that generates an asymmetric Gabor filter.

Traditionally, the Gabor filter has been proposed as a model to describe the receptive field of simple cells, and successfully predicts the responses of cortical neurons of monkeys and cats (Marcelja, 1980; Kulikowski et al., 1982; Daugman, 1985; Field and Tolhurst, 1986; Jones and Palmer, 1987b). The Gabor model is optimal in terms of minimizing the uncertainty associated with localizing a signal in both space and SF (Marcelja, 1980; Daugman, 1985), and therefore can create a sparse representation of natural images. The modified model we present here keeps the basic characteristics of the classic model and introduces one additional parameter that accounts for spatial asymmetry.

Although the tilted model succeeds in predicting the observed responses, there is still no experimental study in mice that mapped the RF of cells directly, and accordingly characterized asymmetry based on observations. However, a study in cats (Jones and Palmer, 1987a) showed that the 2D response profile of simple cells is not necessarily Cartesian separable due to a relative displacement of the subfields, and that within a given receptive field, subfields need not be the same length. In addition, visual inspection of examples of cells from studies conducted in mice also revealed some displacement between the ON and OFF subfields of the RF of cells (Bonin et al., 2011; Ko et al., 2013; Cossell et al., 2015). However, Bonin et al. (2011) used wavelet stimuli to map the RF of cells (Selesnick et al., 2005), which are spatially symmetric by nature, while Ko et al. (2013) and Cossell et al. (2015) presented natural images but used a regularized pseudoinverse method to estimate the RF of cells (Smyth et al., 2003), which introduces a two-dimensional smoothness constraint on the RF and might bias the RF structure toward more symmetric organization.

### Functional role of asymmetry in neuronal representations

Breaking the symmetry of the receptive field of neurons has been previously reported in the retina and the hippocampus. In the retina, asymmetry is observed between the sizes of the circular receptive fields of ON and OFF ganglion cells (Chichilnisky and Kalmar, 2002), or between the relative dendritic field size of parasol and midget cells (Dacey and Petersen, 1992), or in the asymmetric adaptation of ON and OFF ganglion cells to photopic (day) and scotopic (night) conditions (Pandarinath et al., 2010).

Hippocampal place cells exhibit an asymmetry in the spatial arrangement of their place fields. That is, as the animal moves through the receptive field of a cell, the firing rate modulates asymmetrically, where at the start of the place field, the firing rate is low, and at the end of the field, the firing rate is higher. The asymmetry increases as a function of familiarity (Mehta et al., 2000). This asymmetry also exists at the level of subthreshold excitatory inputs to place cells, and has been proposed to arise from a change in the balance between inhibitory inputs at the pyramidal cell soma and increases in dendritic excitation (Harvey et al., 2009). This place cell asymmetry represents a prospective coding scheme, where the increased firing skewness early in the receptive field signals upcoming place field center. The network mechanisms of this asymmetry however, are not completely known.

Furthermore, asymmetry in the visual cortex might play an important role in contour integration, which is in line with the gestalt principles of perceptual grouping (Metzger, 2006), specifically the law of “good continuation” (Field et al., 1993). Since neighboring neurons share RF subfields (Smith and Häusser, 2010), then the integration of local populations of neurons, some of which exhibit spatial RF asymmetry, may be the basis for cortical representation of spatial continuity in curved shapes.

## Synthesis

Reviewing Editor: Leonard Maler, University of Ottawa<br />Decisions are customarily a result of the Reviewing Editor and the peer reviewers coming together and discussing their recommendations until a consensus is reached. When revisions are invited, a fact-based synthesis statement explaining their decision and outlining what is needed to prepare a revision will be listed below. The following reviewer(s) agreed to reveal their identity: Robert Shapley. For some manuscripts transferred from JNeurosci, a decision is made to accept the manuscript without peer review and a brief statement to this effect will follow.

The first reviewer now finds this an excellent contribution to the literature in this field. This reviewer does have some fairly minor issues that remain to be addressed but does not require a re-review.

As Editor, I will make sure that these remaining issues have been corrected in your next submission.

The second reviewer has not responded to repeated requests to re-review. I took it upon myself to read the Ms and carefully check for the modifications made in response to this reviewers detailed comments.

I find that all the second reviewers important concerns have been addressed; in particular, I was pleased to see the critical issues of statistical analyses were addressed and the requested analysis added.

I therefore consider that this Ms is now suitable for publication after the minor issues of the first reviewer have been taken care of.
